# Can museum egg specimens be used for proteomic analyses?

**DOI:** 10.1186/1477-5956-8-40

**Published:** 2010-07-14

**Authors:** Steven J Portugal, Helen J Cooper, Cleidiane G Zampronio, Laine L Wallace, Phillip Cassey

**Affiliations:** 1 The Centre for Ornithology, School of Biosciences, College of Life and Environmental Sciences, The University of Birmingham, Edgbaston, Birmingham, B15 2TT, UK; 2 School of Biosciences, College of Life and Environmental Sciences, The University of Birmingham, Edgbaston, Birmingham, B15 2TT, UK; 3 Functional Genomics and Proteomics Laboratory, School of Biosciences, College of Life and Environmental Sciences, The University of Birmingham, Edgbaston, Birmingham, B15 2TT, UK

## Abstract

**Background:**

Mass spectrometry and proteomic analyses have become powerful tools for the analysis of proteins and peptides. Investigation of proteins contained in the various layers of the avian eggshell has focused entirely on domesticated species. It has been widely assumed that this existing research can inform the study of wild bird species despite the fact that the vast majority of the diversity in avian species (~95%) exists outside the Orders to which domestic and poultry species belong. Museum collections offer a potentially valuable source of material for studying composition of wild avian eggshell matrix proteins. We used museum and fresh eggshells of common quails *Coturnix coturnix *to compare the protein composition of their organic matrices. Four eggs of domestic chickens were analysed simultaneously as a control for comparison to the fresh and museum quail eggs. The determination of the proteins was carried out using enzymatic cleavage followed by high-performance mass spectrometry.

**Results:**

We found that some of the expected key eggshell proteins (3 out of 11) were not present in the samples of museum quail egg. These proteins were either entirely absent from the museum eggs or the technique was unable to detect them. There was no pattern in the absent proteins in the sense of protein function or where they are located within the eggshell.

**Conclusion:**

We conclude it is likely that such studies on museum specimens using a proteomic approach will be limited in coverage of proteins and may, therefore, be misleading.

## Background

Mass spectrometry and proteomic analyses have become powerful tools for the analysis of proteins and peptides [1]. The recent elucidation of the chicken genome has provided the opportunity to apply this technique to identify and then categorise the matrix proteome of the chicken eggshell [2,3]. The avian eggshell is a highly regulated crystalline biocomposite ceramic composed of multiple layers, predominantly the trigonal phase of calcium carbonate (CaCO_3_), known as calcite [3]. Eggshells provide a number of important functions such as protection against physical damage and microbial contamination, regulation of gas and water exchange during embryogenesis, and provision of calcium for embryonic development [3,4]. The general structure of the eggshell consists of the innermost membrane, a continuous calcified layer, and the outermost cuticle [2,4], and thus far, over 520 proteins have been identified from these three layers [2] by use of a proteomics approach.

With few exceptions, most studies on eggshell proteins have been associated with the commercial poultry industry or other domesticated species [5,6]. For example, Panheleux et al. [6] compared the biochemical characteristics of eggshells from seven domesticated species of birds. Their study showed the presence of common matrix components in the various domestic avian species, supporting the notion of universality of their distribution across species. However, their study also demonstrated some particularities, with not all of the domesticated species having the key eggshell proteins that were the focus of the study [6]. It had been widely assumed that this existing research can inform the study of wild bird species despite the fact that the vast majority of the diversity in avian species (~95%) exists outside the Orders to which domestic and poultry species belong. While the general structure of the eggshell is similar across all bird species, the thickness, form and size of the whole eggshell and its mineralised microstructure all vary between species [7]. Moreover, the organic and organomineral content of the eggshell matrix that is believed to regulate eggshell mineralization differs between the domestic species that have been studied so far. For example, non-proteomic approaches have shown that the eggs of ostriches (*Struthio camelus*), emus (*Dromaius novaehollandiae*), rheas (*Rhea americana*) and domestic ducks (*Anas p. domestica*) contain species-specific proteins that are not found in poultry but are thought to be homologous in function, at least, to a chicken protein [2,8,9]. Therefore, the little evidence available suggests that eggshell proteins may be present in different quantities and different forms in different species.

To answer key ecological questions as to why the structure and composition of the eggshell differ among species, it is thus vital to be able to characterise the eggshell matrix components from a number of bird species from a wide range of Orders. As a result of persecution, collecting fresh wild bird eggs was made illegal in the United Kingdom in 1954 (*Wild Birds Protection Act*). Therefore, museum egg specimens are an important repository of historical samples [10]. As such, museum egg specimens could provide an unprecedented opportunity to sample proteins from a wide range of wild bird species from a multitude of Orders.

The sampling of museum eggs has been made possible through a collaboration between the Centre for Ornithology, University of Birmingham (UK) and the Natural History Museum (NHM, Tring, UK), which made a limited number of data-poor eggs available for destructive analysis. The NHM collection is believed to be the most comprehensive in the world with an estimated one million eggs [11]. However, it is not known if the eggshell proteins will degrade over time, and thus not be present in museum eggs of unknown age. That occurrence would make it difficult to draw conclusions as to whether a protein was simply never present in the eggshell, or absent through temporal degradation. Simple protein and DNA examination has been conducted on fossilised dinosaur eggshells [12,13], but no such work has yet been conducted on museum bird eggs which have potentially been treated with detergents and chemicals. Therefore, the aim of this study was to use both fresh and museum eggs of common quails *Coturnix coturnix *to investigate how the protein composition of the two eggshell matrices compared, and to ascertain whether museum eggs are suitable candidates for study using modern proteomic techniques.

## Results

Four museum and four fresh quail eggs were analysed. Each egg came from a different clutch. For the four museum eggs, location and date of collection are; 1925 (Cornwall), between 1961-1963 (Aberdeen), 1901-1910 (England, exact location unknown) and 1911-1930 (North Yorkshire). To ensure the protein extraction procedure and protocol were effective at detecting the eggshell proteins consistently, four eggs of domestic chickens were analysed simultaneously as a control for comparison to the fresh and museum quail eggs. This ensured that any proteins not detected in the museum eggs was not a result of a methodological artefact. The fresh eggs of the chickens and quails were commercially obtained from local retailers. Four replicates were studied from each egg. As the four museum eggs were collected in the United Kingdom, it is assumed they all belong to the *Coturnix coturnix *nominate race. The four fresh quail eggs were from a quail breeder in north-Worcestershire (UK), and were also of the *Coturnix coturnix *nominate race.

Two protocols were used for protein extraction. The preparation of the various insoluble layers followed the previously published method, described in detail in Mann et al. [2]. Briefly the eggshells were treated with 5% EDTA and then washed extensively with distilled water to facilitate mechanical removal of the membrane. In protocol one, the crushed calcified eggshell layer was demineralised with 10% acetic acid. Acid-insoluble material was removed by centrifugation and the supernatant, containing the soluble matrix, was dialysed against 5% acetic acid and lyophilised. A second extraction protocol was based loosely on that of Mann et al [14]. The shell fragments were dissolved in 8 M urea, the solution was diluted to 2 M urea with 0.2 M ammonium bicarbonate, and the samples lyophilised. As we were not attempting to quantify proteins, sub samples of the shells were used.

After lyophilisation and reconstitution in a known volume, an OD (optical density) 280nm was performed to ascertain protein concentration. Fixed volumes were used for digestion as opposed to protein concentration. The samples were dried down to 0.5 ml, and the pH adjusted with 5M NaOH to pH 7.0. Added to this solution was 50 μl 10 mM dithiothreitol (DTT), prior to the samples being incubated at 60°C for 30 mins. Samples were then cooled to room temperature for a further 5-10 mins and cysteines alkylated by addition of 50μl 50mM iodoacetamide, mixed and incubated at room temperature in the dark for 45 mins. For the first protocol, 50 μl of trypsin gold (Promega, Southampton, Hampshire, UK, 6 ng/μl) was subsequently added to the samples, which were then incubated at 37°C overnight (*c*. 14 hours). For protocol two, 4 μl of lysyl endopeptidase was added to the solution, and left for 14 h at 23°C. The reaction mixture was then diluted to 2 M urea and trypsin added as above. The peptides were extracted and washed using millipore C18 ZipTips. Briefly, tips were prepared by pre-wetting in 100% acetonitrile and rinsed in 2 × 10 μl 0.1% formic acid. Samples were repeat pipetted throughout the volume of the samples five times. The tip was then washed with 3 × 10μl 0.1% formic acid to remove excess salts and undigested protein before elution of peptides with 10-20μl of 50% acetonitrile/water/0.1% formic acid. Samples were dried down to remove the acetonitrile, and then re-suspended in 0.1% formic acid solution in distilled water. All chemicals were purchased from Sigma (Gillingham, Dorset, UK) or Fisher Scientific (Loughborough, Leicestershire, UK). A 7 Tesla LTQ FT ultra mass spectrometer (ThermoFisher Scientific, Germany) were used to perform data-dependent scanning [15]. Data acquisition was controlled by Xcalibur 2.0 software. The mass spectrometer alternated between a full FT-MS scan (m/z 380 - 2000) and subsequent collision-induced dissociation (CID) MS/MS scans of the three most abundant ions. Survey scans were acquired in the ICR cell with a resolution of 100 000 at m/z 400. Precursor ions were isolated and subjected to CID in the linear ion trap. Collision activation for the experiment was performed in the linear trap using helium gas at collision energy normalized to precursor m/z of 35% and *q*_excite _= 0.25. The width of the precursor isolation window was 2 m/z and only multiply-charged precursor ions were selected for MS/MS.

**Figure 1:**
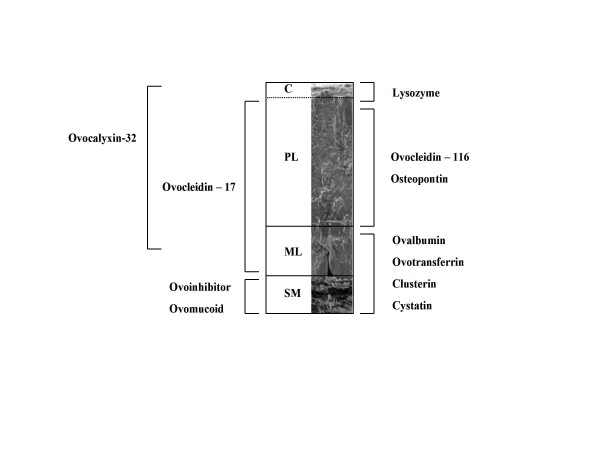
**Distribution of protein types in the avian eggshell**. Differential localised pattern for eggshell matrix proteins within the avian eggshell. Data from different studies (see Hincke et al [3]) are summarised to demonstrate the localisation of the eggshell proteins through the 3 main layers; PL (pallisade layer), ML (mamillary layer) and SM (shell membrane), plus the C (cuticle). The scanning electron microscope image is taken from Hincke et al [3].

Based on Mann et al. [2,16], 11 key eggshell proteins were selected to test for their presence in both fresh and museum quail egg specimens (Fig. 1, Additional File 1). Database searches (Swissprot and NCBInr) were conducted using the MASCOT (Matrix Science, London, UK). Variable modifications were N-acetyl and oxidation (M). The peptide tolerance was 5 ppm and the MS/MS tolerance was 0.5Da. One missed cleavage was allowed. BLAST analysis of identified proteins was performed with the programme provided by the NBCI against the non-redundant database for all organisms. Protein identifications provided by MASCOT software were accepted if the data set contained at least two peptides with a MASCOT score > 25 at *P *> 0.01. Subsequent reverse searches of all data showed the number of false positives to be less than 1%.

Peptide sequences and identified proteins were very similar within each species, and as such, the data presented in Table 1 is for one egg of each of the species and samples (chicken, fresh quail, museum quail). The criteria for which egg is presented, was that it contained all peptide sequences that were present in the other three eggs for each species, plus any additional peptides that were identified in that egg alone. For each of the four runs, all 11 of the key eggshell proteins were identified in the fresh eggs of chickens and quails (Table 1, Additional File 2). The four museum eggs were consistent in their results, with ovocalyxin-32, ovoinhibitor and ovotransferrin consistently being totally absent (Table 1). Cystatin and ovocleidin-17 were only identified in the museum quail eggs using the urea extraction protocol, all other proteins were identified using both urea and acetic acid techniques. In the chicken, ovalbumin, lysozyme and ovocleidin-116 had the highest Mascot scores. For both the fresh and museum quails, the three proteins with the highest Mascot scores were ovomucoid, ovalbumin and ovocleidin-116 (Table 1).

**Table 1:** Proteins detected in fresh chicken and quail eggs in comparison to museum stored quail eggs of unknown age.

	Chicken				Fresh Quail				Museum Quail			
Protein	Present	No. Pept.	Mascot	% Cov.	Present	No. Pept.	Mascot	% Cov.	Present	No. Pept.	Mascot	% Cov.
**Clusterin**	✓	7	201	23	✓	5	145	12	✓	1	39	1
**Cystatin**	✓	5	175	39	✓	5	134	31	✓*	1	40	9
**Lysozyme**	✓	12	227	80	✓	11	315	75	✓	5	5	34
**Osteopontin**	✓	1	59	5	✓	2	48	5	✓	2	61	5
**Ovalbumin**	✓	17	1997	78	✓	17	1053	42	✓	9	378	20
**Ovocalyxin-32**	✓	3	61	44	✓	2	129	4	✗	-	-	-
**Ovocleidin-17**	✓	7	434	54	✓	2	82	33	✓*	2	85	11
**Ovocleidin-116**	✓	24	2017	44	✓	18	1214	42	✓	5	308	12
**Ovoinhibitor**	✓	3	139	9	✓	3	38	10	✗	-	-	-
**Ovomucoid**	✓	3	151	25	✓	14	497	88	✓	7	247	38
**Ovotransferrin**	✓	24	1036	43	✓	2	64	3	✗	-	-	-

A tick mark (✓) denotes presence and a cross (✗) absence. Number of unique peptides is abbreviated to "No. Pept.". Mascot refers to the Mascot score. Further information on peptide sequences is located in Additional File 2. The asterix (*) refers to proteins that were detected only through the urea extraction technique.

## Discussion

The concurrent runs of the fresh eggs of chickens and quails demonstrate the technique used was sufficient to identify the 11 key eggshell proteins in fresh eggs, suggesting those not detected in the museum specimens were either no longer present in the shell or were undetectable using this proteomic approach. Osteopontin was found in the least frequency from a coverage perspective (Additional File 2), but again, was present in all three species eggs suggesting number of peptides and % coverage is not an issue relating to egg storage or time. Ovocleidin-17 was detected in quail eggs for the first time and was identified in both the fresh and museum specimens in the present study (see Additional File 3 for spectra). Panheleux et al [[6], see also [17]] had detected a band of 17 kDa through Western blotting when stained with Coomassie blue suggesting the possible presence of ovocleidin-17 in quails, which the current study has subsequently confirmed. Prior to storage, museum eggs can be chemically treated. The collection we were working with was provided by private collectors, who may use a combination of different mechanical and chemical techniques to remove egg contents. This treatment, plus the potential accumulation of exogenous particulates in storage may affect degradation of proteins [18]. However, if this was to occur, it would be expected to happen in either the cuticle and palisade layer (PL) or the membrane (SM) of the eggshell. A 'missing' protein in the museum eggs such as ovotransferrin, for example, is predominantly found in the mammillary layer (ML), however (Fig. 1). Also, egg collectors typically prefer eggs that have been freshly laid, and will therefore not require extensive chemical treatment, as the contents of the egg will be liquid and easily removed through blowing [19].

A review of techniques used to remove embryos, blow eggs and treat museum eggshells found that pepsin, trypsin and wine vinegar were the most damaging to the eggshell [19]. We are unable to know exactly what treatment the museum quail eggs in the study received, particularly prior to admittance to the museum collection. From a proteomics perspective, trypsin and wine vinegar would be the most problematic. Wine vinegar would cause acid hydrolysis of the proteins. If the eggshells had been treated with trypsin or pepsin, it is not implausible that proteins may have been partially digested in the preparation process. This partial digestion may also cause protein folding, which could prevent the trypsin from acting effectively in the present study, and result in the proteins not being detected. However, unless different proteins respond differently to partial trypsin digestion, there seems no basis why some proteins would have degraded and some not. Again, this effect would be predicted to be most prevalent on the outside layers such as the PL and SM, yet most of the proteins absent from the museum eggs reside in the ML. Bada et al. [20] noted that in prehistoric dinosaur bone, certain proteins didn't degrade as fast as expected because of their close association with bone material. Other factors such as environmental temperature, humidity and the type of matrix in which the molecules are contained are also known to affect the extent of protein degradation [21]. In general, however, the absence of no clear pattern of missing proteins with respect to the layer they are located within the shell (Fig. 1) suggests factors such as storage temperature did not affect protein degradation of one specific layer of the eggshell differently. Therefore, it is likely that degradation of protein in the eggshell is a function of time, rather than storage conditions, at least for the eggs at the Natural History Museum. There are other potential problems for future proteomic work on both fresh and museum eggshells. One is the lack of sequences for more exotic species, which will inevitably lead to missed proteins or mis-identification. This in part will be why all proteomic studies on avian eggshells thus far have focused on domesticated species. The use of data-poor museum eggshells could also present concerns over the provenance of the eggs used, and potentially limit the conclusions that can be drawn from research on such samples.

## Conclusion

In summary, we found that some of the expected proteins (3 out of 11) were entirely absent from samples of museum quail egg. Although the availability of museum eggs is a potential source for the comparative study of proteins using modern proteomic techniques, we caution that such studies will likely be limited in their coverage of proteins and may therefore be misleading.

## Competing interests

The authors declare that they have no competing interests.

## Authors' contributions

SJP executed all experiments, performed the data analysis and wrote the manuscript. HJC and PC assisted with design and interpretation of the experiments and advised on the study. CGZ and LLW conducted sample preparation and analysis. All authors read and approved the final manuscript.

## Supplementary Material

Additional file 1 **Eggshell Protein Types**. Summary of the function of the key eggshell proteins. Click here for file

Additional file 2 **Peptide Sequences Summary Table**. Tables showing all peptides sequences identified for the 11 key eggshell proteins, from fresh chicken eggs, and museum and fresh quail eggs. Click here for file

Additional file 3 **Spectra for Ovocleidin-17**. Full spectra for ovocleidin-17 from both fresh and museum quail eggs. Click here for file
