# Supplementary material for: Can museum egg specimens be used for proteomic analyses?
Source: Proteome Sci. 2010 Jul 14;8:40. doi: 10.1186/1477-5956-8-40 (PMC2927511; doi:10.1186/1477-5956-8-40)
Supplement: Additional file 1 — Eggshell Protein Types. Summary of the function of the key eggshell proteins. [file 1477-5956-8-40-S1.DOC]

**Portugal et al Additional File 1: Eggshell Protein Types**

Detailed below are the key eggshell proteins discussed in the main body of the manuscript, with a brief description of their primary function.

Ovomucoid inhibits proteolytic enzymes (i.e. trypsin inhibitors), which are important for microbial growth [1]. Lysozyme is a bacteriolytic enzyme which catalses the hydrolysis of beta-1,4-glycosidic bonds of polysaccharides, thereby compromising the peptidoglycan layer of bacteria cells walls [2]. Ovotransferrin is a glycoprotein comprised of two domains that each reversibly bind one molecule of iron and thereby impede growth of bacteria by limiting levels of this essential element [3]. Ovoinhibitor acts in a similar fashion to ovomucoid, by inhibiting trypsin, chymotrypsin, subtilisin and elastin [1]. Several eggshell-bound components and proteins which show antimicrobial properties have been demonstrated to be capable of reducing populations of *Salmonella enterica, Escherichia coli* and *Listeria monocytogenes* [4]. Ovalbumin is a noninhibitory member of the serpin family [1]. The eggshell specific proteins are comprised of ovocalyxin-32 (similar to mammal carboxypeptidase inhibitor latexin), ovocleidin-17 (lectin-like phophoprotein) and ovocleidin-116 (dermatan sulphate proteoglycan) [1, 2]. Cystatin is a cysteine protease inhibitor [1]. Clusterin is a disulfide-linked heterodimeric protein, involved in the clearance of cellular debris and apoptosis and also acts as a protein chaperone [1, 2]. Osteopontin is a glycoprotein, also found in bone [5]. Of these 11 eggshell proteins, seven have been identified as phosphoproteins (clusterin, cystatin, ovalbumin, ovocalyin-32, ovocleidin-17, ovocleidin-116 and osteopontin) [6].

[1] Kovaco-Nolan, J., Phillips, M. and Mine, Y. *J. Agric. Food Chem.* 2005, *53*, 8421-8431.

[2] Jolles, P. and Jolles, J. *Mol. Cell. Biochem*. 1984, *63*, 165-189.

[3] Valenti, P., De Stasio, A., Mastromerino, P., Sinibaldi, L. and Orsi, N. *FEMS Microbiol. Lett.* 1981, *10*, 77-79.

[4] Poland, A. L. and Sheldon, B. W. *J. Food. Prot.* 2001, *64*, 486-492.

[5] Mikšĭk, I., Eckhardt, A., Sedláková, P. and Mikulikova, K. *Connect. Tissue Res.* 2007, *48*, 1-8.

[6] Mann, K., Olsen, J. V., Maćek, B., Gnad, F. and Mann, M. *Proteomics.* 2007, *7*, 106
