# Supplementary material for: Can museum egg specimens be used for proteomic analyses?
Source: Proteome Sci. 2010 Jul 14;8:40. doi: 10.1186/1477-5956-8-40 (PMC2927511; doi:10.1186/1477-5956-8-40)
Supplement: Additional file 2 — Peptide Sequences Summary Table. Tables showing all peptides sequences identified for the 11 key eggshell proteins, from fresh chicken eggs, and museum and fresh quail eggs. [file 1477-5956-8-40-S2.DOC]

| **Protein**  **Portugal et al, Additional File 2, Chicken:** Peptide sequences for key eggshell proteins, obtained by acetic acid extraction and trypsin digestion on fresh domestic chicken eggs. | **Mascot Score** | **No. Unique peptides** | **Sequence Coverage** | **Sequence** |
| --- | --- | --- | --- | --- |
| **Clusterin** | 201 | 7 | 23% | R.EAFVPPVQR.V |
|  |  |  |  | R.RFEDLEER.F |
|  |  |  |  | R.SSPFSIWVNGER.I |
|  |  |  |  | R.EILAVDCSQTDPVQSQLR.E |
|  |  |  |  | R.TPPFGGFR.E |
|  |  |  |  | R.EAFVPPVQR.V |
|  |  |  |  | R.EQFEDALR.L |
| **Cystatin** | 175 | 5 | 39% | R.LLGAPVPVDENDEGLQR.A |
|  |  |  |  | K.SSGDLQSCEFHDEPEMAK.Y |
|  |  |  |  | K.YILQVEIGR.T |
|  |  |  |  | R.ALQFAMAEYNR.A |
|  |  |  |  | K.YTTCTFVVYSIPWLNQIK.L |
| **Lysozyme** | 227 | 12 | 80% | R.GYSLGNWVCAAK.F |
|  |  |  |  | K.FESNFNTQATNR.N |
|  |  |  |  | K.IVSDGNGMNAWVAWR.N |
|  |  |  |  | R.NLCNIPCSALLSSDITASVNCAK.K |
|  |  |  |  | R.HGLDNYR.G |
|  |  |  |  | R.CELAAMK.R |
|  |  |  |  | R.WWCNDGR.T |
|  |  |  |  | K.GTDVQAWIR.G |
|  |  |  |  | R.NTDGSTDYGILQINSR.W |
|  |  |  |  | R.CELAAAMKR.H |
|  |  |  |  | K.IVSDGNGMNAWVAWR.N |
|  |  |  |  | K.FESNFNTQATNRNTDGSTDYGILQINSR.W |
| **Osteopontin** | 59 | 1 | 5% | R.GDNAGRGDSVAYGFR.A |
| **Ovalbumin** | 1997 | 17 | 78% | K.IKVYLPR.M |
|  |  |  |  | R.ADHPFLFCIK.H |
|  |  |  |  | K.HIATNAVLFFGR.C |
|  |  |  |  | R.YPLILPEYLQCVK.E |
|  |  |  |  | K.AFKDEDTQAMPFR.VK.LTEWTSSNVMEER.K |
|  |  |  |  | R.GGLEPINFQTAADQAR.E |
|  |  |  |  | K.ISQAVHAAHAEINEAGR.E |
|  |  |  |  | M.GSIGAASMEFCFDVK.E |
|  |  |  |  | R.ELINSWVESQTNGIIR.N |
|  |  |  |  | K.ELYRGGLEPINFQTAADQAR.E |
|  |  |  |  | R.DILNQITKPNDVYSFSLASR.L |
|  |  |  |  | R.VTEQSKPVQMMYQIGLFR.V |
|  |  |  |  | R.LYAEERYPILPEYLQCVK.E |
|  |  |  |  | R.VTEQSKPVQMMYQIGLFR.V |
|  |  |  |  | R.NVLQPSSVDSQTAMVLVNAIVFK.G |
|  |  |  |  | R.FDKLPGFGDSIEAQCGTSVNVHSSLR.D |
|  |  |  |  | K.VHHANENIFYCPIAIMSALAMVYLGAK.D |
| **Ovocalyxin-32** | 61 | 3 | 44% | R.FYEYLQHQK.K |
|  |  |  |  | K.QSTEHTGYLLAQVSSVK.Q |
|  |  |  |  | K.FIVLLHEIPTQQINYCHMYLVWTLGHPIR.V |
| **Ovocleidin-17** | 434 | 7 | 54% | R.LLAELLNASR.G |
|  |  |  |  | R.WGPGSHLAAVR |
|  |  |  |  | R.VWIGLHRPAGSR.S |
|  |  |  |  | R.CAALRDEEAFTSWAARPCTER.N |
|  |  |  |  | R.ELSWSR.A |
|  |  |  |  | R.WSDGTAPR.F |
| **Ovocleidin-116** | 2017 | 24 | 44% | R.VQQEVAPAR.G |
|  |  |  |  | R.LGGLTEMEHSR.Q |
|  |  |  |  | R.GVVGGMVVPEGHR.A |
|  |  |  |  | R.GSTVAGGFAHLHR.G |
|  |  |  |  | R.GNCPGQHQILLK.G |
|  |  |  |  | R.TQPEVASAPSTVGK.A |
|  |  |  |  | R.ARGNCPGGQHQILLK.G |
|  |  |  |  | R.ARTQPEVASAPSTVGK.A |
|  |  |  |  | R.GQDGETHISPEDEVK.I |
|  |  |  |  | R.LGQAARPEVAPAPSTGGR.I |
|  |  |  |  | K.EEGDHQGTIHGHWLGK.V |
|  |  |  |  | K.VDGEAPGQGVGSSHVPEDK.D |
|  |  |  |  | R.DPWVWGSAHPQAQHTR.G |
|  |  |  |  | K.KEEGDHQGTIHGHWLGK.V |
|  |  |  |  | R.VWPGAAPAPGVARPAPSK.A |
|  |  |  |  | R.GTASSGLTTGDCSTAASTPSR.K |
|  |  |  |  | R.AQQEVAPVPSMVVETVAPER.H |
|  |  |  |  | R.ARVWPGAAPAPGWGVARPAPSK.A |
|  |  |  |  | R.GGRGTASSGLTTGDCSTAASTPSR.K |
|  |  |  |  | R.NRAQQEVAPVPSMVVETVAPER.H |
|  |  |  |  | K.STDVPRDWVWGSAHPQAQHTR.G |
|  |  |  |  | K.EDVHVDTEGIDEFAYIPDVDAVTITR.G |
|  |  |  |  | K.VDGEAPGQGVGSSHVPEDKDSPKPHSITPASKGEGR.A |
|  |  |  |  | K.GAGSEGGSHATVPDQGQAGTMGTGDSAITSVTDSAITSVTK.K |
| **Ovoinhibitor** | 139 | 3 | 9% | K.LEIGSVDCSKYPSTVSK.D |
|  |  |  |  | R.ILSPVCGTDGFTYDNECGICAHNAEQR.T |
|  |  |  |  | K.DGTSWVACPR.N |
| **Ovomucoid** | 151 | 3 | 25% | K.DVLVCNK.D |
|  |  |  |  | R.AFNPVCGTDGVTYDNECLLCAHK.V |
|  |  |  |  | K.ELAAVSVDCSEYPKPDCTAEDRPLCGSDNK.T |
| **Ovotransferrin** | 1036 | 24 | 43% | R.ISLTCVQK.A |
|  |  |  |  | K.ATYLDCIK.A |
|  |  |  |  | K.YFGYTGALR.C |
|  |  |  |  | K.DPVLKDLLFK.D |
|  |  |  |  | K.SDFHLFGPPGK.K |
|  |  |  |  | R.WCTISSPEEK.K |
|  |  |  |  | K.GTEFTVNDLQGK.T |
|  |  |  |  | R.SAGWNIPIGTLLHR.G |
|  |  |  |  | K.FFSASCVPGATIEQK.L |
|  |  |  |  | R.IQWCAVGKDEK.S |
|  |  |  |  | R.LCQLCQGSGGIPPEK.C |
|  |  |  |  | R.NAPYSGYSGAFHCLK.D |
|  |  |  |  | K.EFLGDKYTVISSLK.T |
|  |  |  |  | R.GAIEWEGIESGSVEQAVAK.F |
|  |  |  |  | K.TCNPSDILQMCSFLEGK |
|  |  |  |  | R.ECNLAEVPTHAVVRPEK.A |
|  |  |  |  | R.EGTCNFDEYFSEGCAPGSPPNSR.L |
|  |  |  |  | R.WSVVSNGDVECTVVDETKDCIIK.I |
|  |  |  |  | K.GDVAFIQHSTVEENTGGK.N |
|  |  |  |  | R.NAPYSGYGAFHCLK.D |
|  |  |  |  | K.RVPSLMDSQLYLGFEYYSAIQSMR.K |
|  |  |  |  | K.LKPIAEVYEHTEGSTTSYYAVAVVK.K |
|  |  |  |  | K.SDFHLFGPPGK.K |
|  |  |  |  | K.NLQMDDFELLCTDGRR.AR.DDNKVEDISWFLSK.A |

| **Protein**  **Portugal et al, Additional File 2, Fresh Quail:** Peptide sequences for key eggshell proteins, obtained by acetic acid extraction and trypsin digestion on fresh quail eggs. | **Mascot Score** | **No. Unique peptides** | **Sequence Coverage** | **Sequence** |
| --- | --- | --- | --- | --- |
| **Clusterin** | 145 | 5 | 12% | R.TPPFGGFR.E |
|  |  |  |  | R.EAFVPPVQR.V |
|  |  |  |  | R.SSPFSIWVNGER.I |
|  |  |  |  | R.EQFEDALR.L |
|  |  |  |  | R.EILAVDCSQTDPVQSQLR.E |
| **Cystatin** | 134 | 5 | 31% | R.LLGAPVPVRENDEGLR.A |
|  |  |  |  | R.LLGAPVPVR.E |
|  |  |  |  | K.YIMEVEIGR.T |
|  |  |  |  | R.ALQFAMAEYNK.A |
|  |  |  |  | R.LLGAPVPVRENDEGLQR.A |
| **Lysozyme** | 315 | 11 | 75% | K.FESNFNTQATNR.N |
|  |  |  |  | R.NTDGSTDYGILQINSR.W |
|  |  |  |  | K.GTDVNAWRIR.G |
|  |  |  |  | K.IVSDVHGMNAWVAWR.N |
|  |  |  |  | R.GYSLGNWVCAAK.F |
|  |  |  |  | R.NLCNIPCSALLSSDITASVNCAK.K |
|  |  |  |  | R.CELAAAMK.R |
|  |  |  |  | R.WWCNDGR.T |
|  |  |  |  | R.CKGTDVNAWIR.G |
|  |  |  |  | K.YQGYSLGNWVCAAK.F |
|  |  |  |  | R.HGLDKYQGYSLGNWVCAAK.F |
| **Osteopontin** | 48 | 2 | 5% | R.GDSVAYGFR.A |
|  |  |  |  | R.GDNAGRGDSVAYFR.A |
| **Ovalbumin** | 1053 | 17 | 42% | R.DILNQITK.Q |
|  |  |  |  | K.AEDTQTIPFR.V |
|  |  |  |  | K.QNDAYSFSLASR.L |
|  |  |  |  | K.HIETNAILLFGR.C |
|  |  |  |  | K.AFKAEDTQTIPFR.V |
|  |  |  |  | R.GGLESVNFQTAADQAR.G |
|  |  |  |  | R.GLINAWVESQTNGIIR.N |
|  |  |  |  | K.ISQAVHAAHAEINEAGR.E |
|  |  |  |  | R.ELINSWVESQTNGIIR.N |
|  |  |  |  | K.LTEWTSSSIMEER.K  R.VTEQESKPVQMMHQIGSFK.V |
|  |  |  |  | R.NILQPSSVDSQTAMVLVNAIAFK.G |
|  |  |  |  | R.ADHPFLFCVK.H |
|  |  |  |  | R.LYAQETYTVVPEYLQCVK.E |
|  |  |  |  | R.ADHPFLCIK.H |
|  |  |  |  | R.YPILPEYLQCVK.E |
|  |  |  |  | R.GGLEPINFQTAADQAR.E |
| **Ovocalyxin-32** | 129 | 2 | 4% | R.GLLSSPTIITGLHLER.S |
|  |  |  |  | R.AMQQVLSDAIIQTGLLEK.H |
| **Ovocleidin-17** | 82 | 2 | 33% | R.LLAELLNASR.G |
|  |  |  |  | R.WGPGSHLAAVR.S |
| **Ovocleidin-116** | 1214 | 18 | 42% | R.VQQEVAPAR.G |
|  |  |  |  | K.HSLPATMTTR.G |
|  |  |  |  | R.LGGLTEMEHSR.Q |
|  |  |  |  | R.GVVGGMVVPEGHR.A |
|  |  |  |  | R.GSTVAGGFAHLHR.G |
|  |  |  |  | R.GNCPGQHQILLK.G |
|  |  |  |  | R.TQPEVASAPSTVGK.A |
|  |  |  |  | R.GQDGETHISPEDEVK.I |
|  |  |  |  | R.LGQAARPEVAPAPSTGGR.I |
|  |  |  |  | K.VDGEAPGQGVGSSHPEDK.D |
|  |  |  |  | R.DPWVWGSAHPQAQHTR.G |
|  |  |  |  | K.KEEGDHQGTIHGHWLGK.V |
|  |  |  |  | R.GTASSGLTTGDCSTAASTPSR.K |
|  |  |  |  | R.AQQEVAPVPSMVVETVAPER.H |
|  |  |  |  | K.STDVPRDPWVWGSAHPQAQHTR.G |
|  |  |  |  | K.EDVHVDTEGIDEFAYIPDVDAVTITR.G |
|  |  |  |  | K.VDGEAPGQGVGSSHVPEDKDSPKPHSHITPASK.G |
|  |  |  |  | K.GAGSEGGSHATVPDQGQAGTMGTGDSAITSVTDSAITSVTK.K |
| **Ovoinhibitor** | 38 | 3 | 10% | R.TLVACPR.I |
|  |  |  |  | K.DGTSWVACPR.N |
|  |  |  |  | R.QFVQVALALCCFADIAFGIEVNCSLYASGIGK.D |
| **Ovomucoid** | 497 | 14 | 88% | K.CDFCNAVVESNGTLTLNHFGK.C |
|  |  |  |  | K.ELAAVSVDCSEYPKPDCTAEDRPLCGSDNK.T |
|  |  |  |  | K.VMVLCNR.A |
|  |  |  |  | R.AFNPVCGTDGVTYDNECLLCAHK.V |
|  |  |  |  | K.VTILCTK.D |
|  |  |  |  | K.DEVVCPDELR.L |
|  |  |  |  | K.DYRPVCGSDNK.T |
|  |  |  |  | K.EQDGECGETVPMDCSR.Y |
|  |  |  |  | K.ELAAVSVDCSEYPKPACPK.D |
|  |  |  |  | R.KELAAVSVDCSEYPKPACPK.D |
|  |  |  |  | R.FPNTTNEEGKDEVVCPDELR.L |
|  |  |  |  | K.CNFCNAVVESNGTLTLNHFGK.C |
|  |  |  |  | R.LICGTDGVTYNHECMLCFYNK.E |
|  |  |  |  | K.DFSFVCGTDGVTYNECMLCAHNVVQGTSVK.K |
| **Ovotransferrin** | 64 | 2 | 3% | K.DPVLKDLLFK.D |
|  |  |  |  | K.DLLFKDLTK.C |

| **Protein**  **Portugal et al, Additional File 2, Museum Quail:** Peptide sequences for key eggshell proteins, obtained by acetic acid extraction and trypsin digestion on museum domestic quail eggs. | **Mascot Score** | **No. Unique peptides** | **Sequence Coverage** | **Sequence** |
| --- | --- | --- | --- | --- |
| **Clusterin** | 39 | 1 | 1% | R.RFEDLEER.F |
| **Cystatin** | 40 | 1 | 9% | R.ALQFAMAEYNK.A |
| **Lysozyme** | 122 | 5 | 34% | R.LGLDNYR.G |
|  |  |  |  | K.NACGIPCSVLLR.S |
|  |  |  |  | R.NTDGSTDYGILQINSR.W |
|  |  |  |  | K.GTDVNAWIR.G |
|  |  |  |  | K.FESNFNTQATNR.N |
| **Osteopontin** | 61 | 2 | 5% | R.GDSVAYGFRA.A |
|  |  |  |  | R.GDNAGRGDSVAYGFR.A |
| **Ovalbumin** | 378 | 9 | 20% | R.GGLEPINFQTAADQAR.E |
|  |  |  |  | R.GGLESVNFQTAADQAR.G |
|  |  |  |  | K.ISQAVHAAHAEINEAGR.D |
|  |  |  |  | K.HIETNAILLFGR.C |
|  |  |  |  | R.ADHPFLFCVK.H |
|  |  |  |  | K.AEDTQTIPFR.V |
|  |  |  |  | R.DILNQITK |
|  |  |  |  | R.DILNQITK.M |
|  |  |  |  | K.VYLPRMK.M |
| **Ovocleidin-17** | 85 | 2 | 11% | R.AESFCR.R |
|  |  |  |  | R.LLAELLNASR.G |
| **Ovocleidin-116** | 308 | 5 | 12% | R.LGQAARPEVAPAPSTGGR.I |
|  |  |  |  | R.GQDGETHISPEDEVK.I |
|  |  |  |  | R.TQPEVASAPSTVGK.A |
|  |  |  |  | R.ARGNCPGQHQILLK.G |
|  |  |  |  | R.GNCPGQHQILLK.G |
| **Ovomucoid** | 247 | 7 | 38% | K.CNFCNAVVESNGTLTLNHFGK.C |
|  |  |  |  | K.ELAAVSVDCSEYPKPACPK.D |
|  |  |  |  | K.ELAAVSVDCSEYPKPDCTAEDRPLCGSDNK.T |
|  |  |  |  | K.CNFCNAVVESNGTLTLSHFGK.C |
|  |  |  |  | K.VMVLCNR.A |
|  |  |  |  | K.VTILCTK.D |
|  |  |  |  | R.AFNPVCGTDGVTYDNECLLCAHK.V |
