# Supplementary material for: Can museum egg specimens be used for proteomic analyses?
Source: Proteome Sci. 2010 Jul 14;8:40. doi: 10.1186/1477-5956-8-40 (PMC2927511; doi:10.1186/1477-5956-8-40)

**Portugal et al, Additional File 3: Spectra for Ovocleidin-17 found in the fresh and museum quail eggs**

Ovocleidin-17

Museum Quail Egg

Peptide sequence: R.LLAELLNASR.G


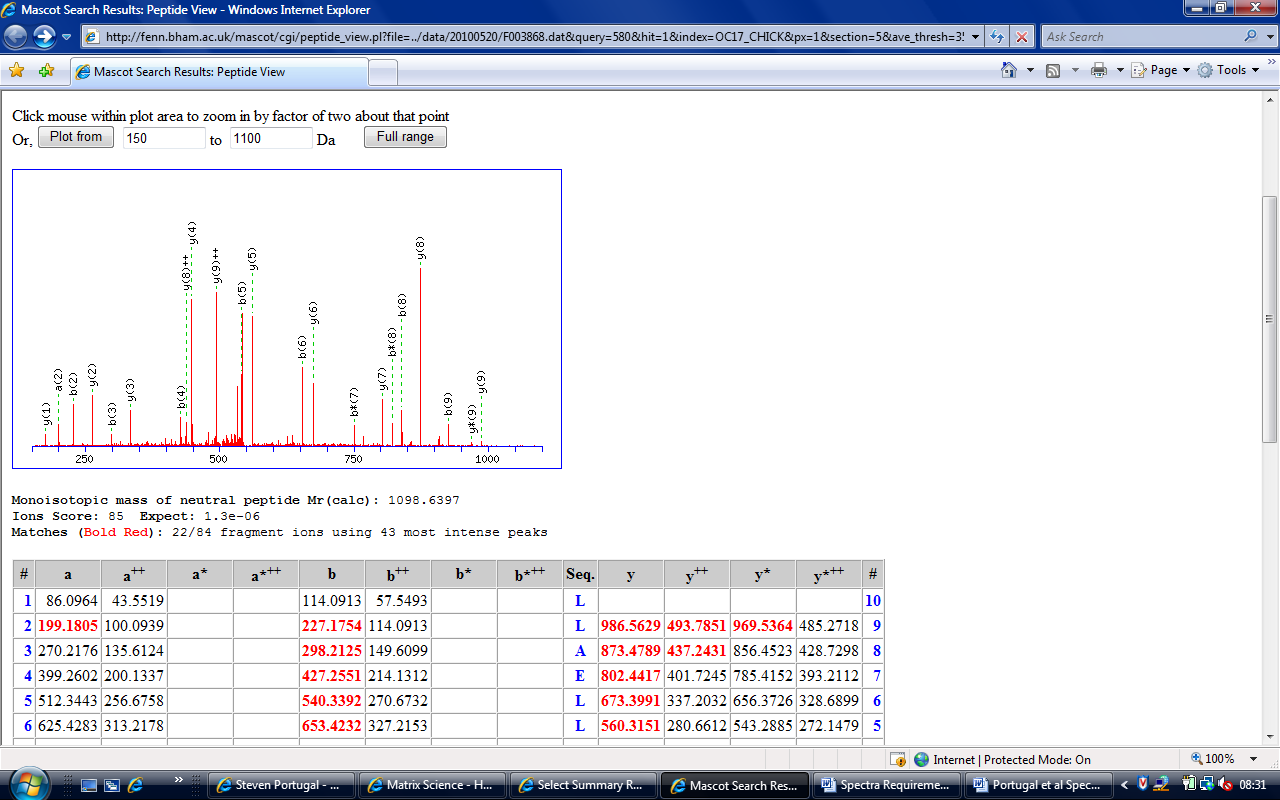


Ovocleidin-17

Fresh Quail Egg

Peptide sequence: R.LLAELLNASR.G


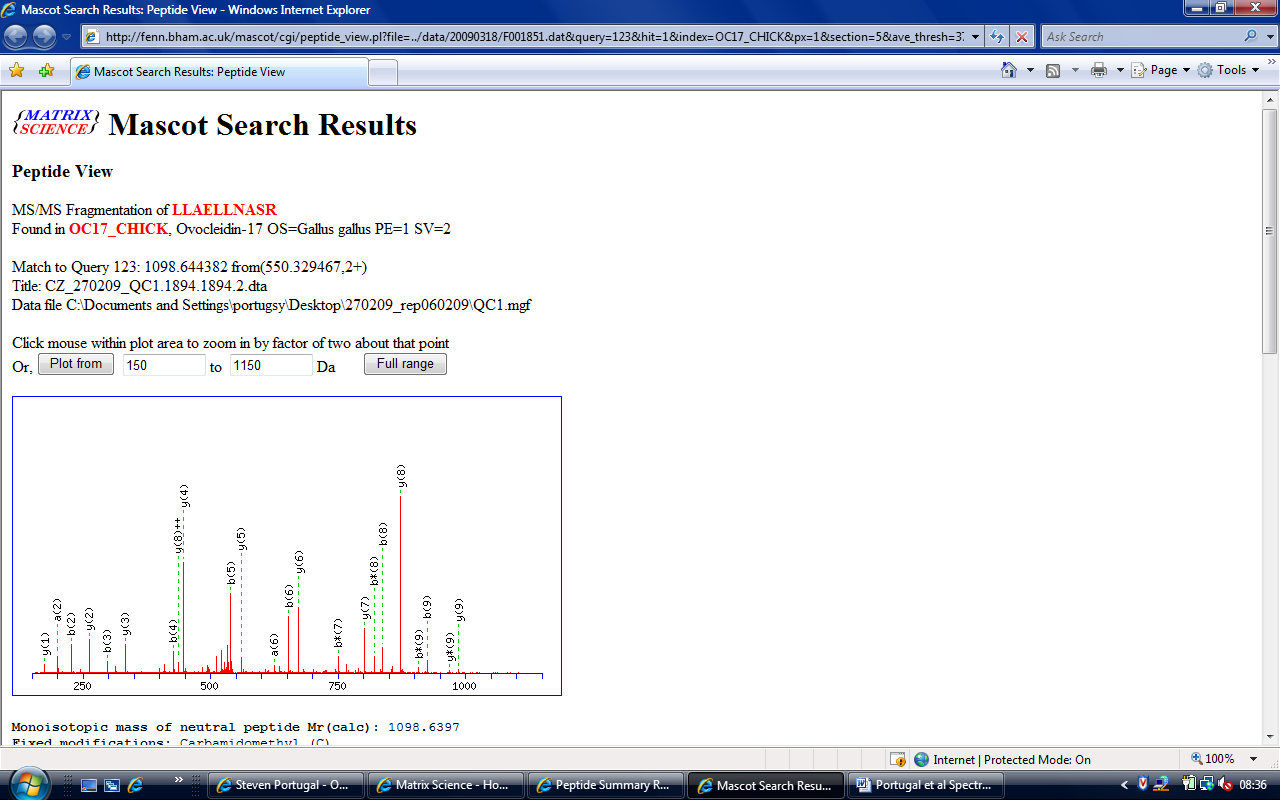


Peptide sequence: R. WGPGSHLAAVR.S


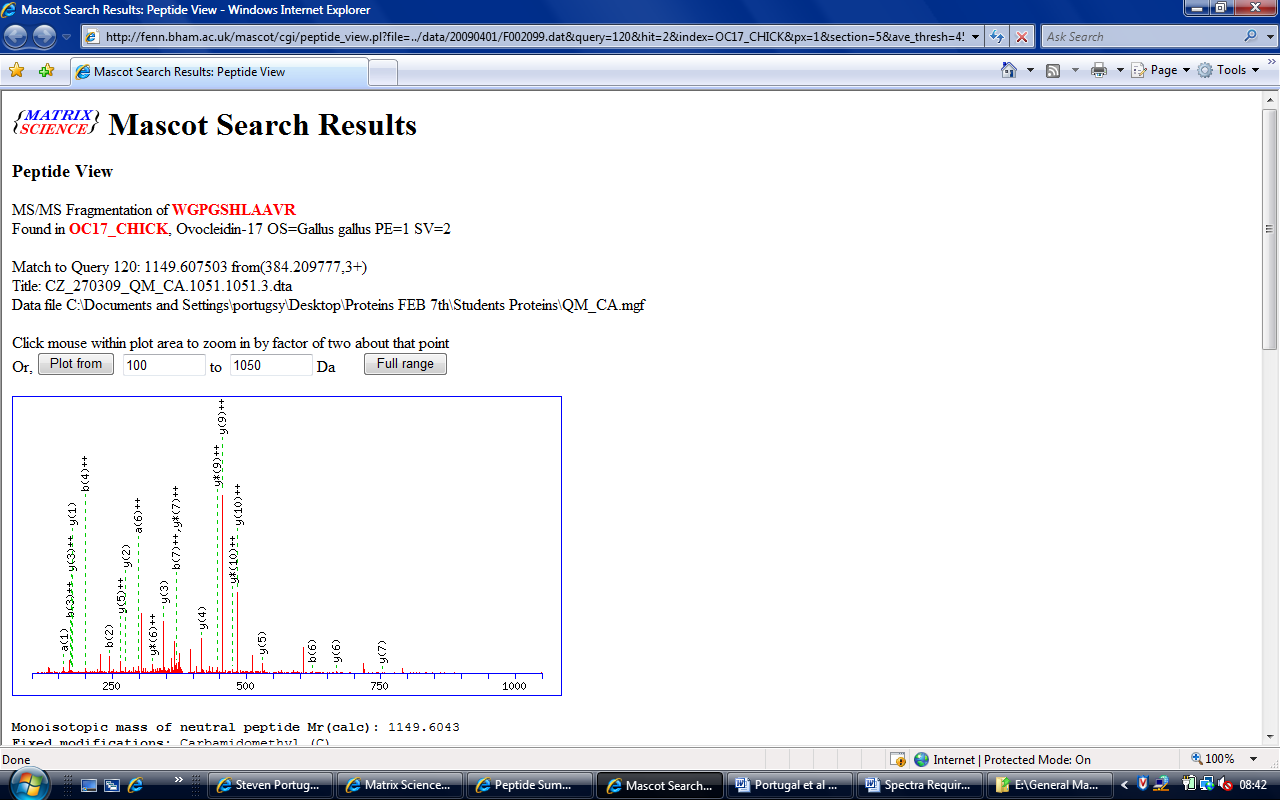

Supplement: Additional file 3 — Spectra for Ovocleidin-17. Full spectra for ovocleidin-17 from both fresh and museum quail eggs. [file 1477-5956-8-40-S3.DOC]
